# 3D‐Printed Breast Prosthesis that Smartly Senses and Targets Breast Cancer Relapse

**DOI:** 10.1002/advs.202402345

**Published:** 2024-09-23

**Authors:** Lu Wang, Chenyang Ye, Xiangjie Xue, Mingjun Xie, Yicheng Zhi, Xiao Feng, Pengcheng Zhao, Jichun Zhou, Mi Mi, Jinrui Li, Qinhao Gu, Ye Zhao, Jiaxin Chen, Yi Zhou, Yanan Xue, Zexin Fu, Liuyi Zhou, Lulu Chen, Lei Pan, Yi Sun, Linbo Wang, Sufan Wu, Yong He, Ji Wang

**Affiliations:** ^1^ Center for Plastic & Reconstructive Surgery Department of Plastic & Reconstructive Surgery Zhejiang Provincial People's Hospital (Affiliated People's Hospital Hangzhou Medical College) Hangzhou 310014 China; ^2^ Department of Medical Oncology Key Laboratory of Cancer Prevention and Intervention The Second Affiliated Hospital of Zhejiang University School of Medicine Cancer Center Zhejiang University Hangzhou 310058 China; ^3^ Department of Surgical Oncology Biomedical Research Center and Key Laboratory of Biotherapy of Zhejiang Province Sir Run Run Shaw Hospital Zhejiang University School of Medicine Hangzhou 310016 China; ^4^ State Key Laboratory of Fluid Power and Mechatronic Systems School of Mechanical Engineering Zhejiang University Hangzhou 310058 China; ^5^ Key Laboratory of 3D Printing Process and Equipment of Zhejiang Province School of Mechanical Engineering, Zhejiang University Hangzhou 310058 China

**Keywords:** 3D‐printed prosthesis, breast reconstruction, drug delivery, relapse treatment

## Abstract

Breast reconstruction is essential for improving the appearance of patients after cancer surgery. Traditional breast prostheses are not appropriate for those undergoing partial resections and cannot detect and treat locoregional recurrence. Personalized shape prostheses that can smartly sense tumor relapse and deliver therapeutics are needed. A 3D‐printed prosthesis that contains a therapeutic hydrogel is developed. The hydrogel, which is fabricated by crosslinking the polyvinyl alcohol with *N1*‐(4‐boronobenzyl)‐*N3*‐(4‐boronophenyl)‐*N1*, *N1*, *N3*, *N3*‐tetramethylpropane‐1,3‐diaminium, is responsive to reactive oxygen species (ROS) in the tumor microenvironment. Specifically, RSL3, a ferroptosis inducer that is loaded in hydrogels, can trigger tumor ferroptosis. Intriguingly, RSL3 encapsulated in the ROS‐responsive hydrogel exerts antitumor effects by increasing the numbers of tumor‐infiltrated CD4+ T cells, CD8+ T cells, and M1 macrophages while reducing the number of M2 macrophages. Therefore, this new prosthesis not only allows personalized shape reconstruction, but also detects and inhibits tumor recurrence. This combination of aesthetic appearance and therapeutic function can be very beneficial for breast cancer patients undergoing surgery.

## Graphical abstract

In this study, a 3D‐printed prosthesis with good biocompatibility, stability, and elasticity is combined with an RSL3@LIPO‐loaded ROS‐responsive hydrogel, which not only optimizes the shape of the breast after surgery in a personalized manner, but also senses tumor, induces ferroptosis in tumor cells, and accumulates the tumor‐suppressive immune cells for exerting therapeutic effect upon tumor recurrence.

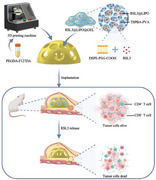

## 1 Introduction

Breast cancer is the most common cancer in women worldwide, accounting for ≈30% of newly diagnosed female malignancies, and the second leading cause of death among cancer patients (11.6%).^[^ ^1^ ^]^ The most common treatment for breast cancer is surgery, which includes whole breast resection and partial breast resection.^[^ ^2^ ^]^ Breast reconstruction is often performed to restore the shape of the breast following surgery. When patients refuse to use autologous flaps due to donor‐site damage, silicone prostheses are the only choice for breast reconstruction. However, even the latest silicone prostheses are designed primarily for patients with whole breast resection. For patients undergoing partial breast resection (for example, one‐third of the breast is resected), surgeons can only suture the remaining incisal edge together, leading to an ugly appearance. Thus far, no suitable prosthesis is commercially available to treat partial breast resection. Recently, 3D printing, an additive manufacturing technique, has been used to manufacture patient‐specific constructs.^[^ ^3^ ^]^ This technique shows great potential for fabricating shape‐personalized prostheses designed for patients with partial breast resection. Nonetheless, the application of 3D‐printed breast prostheses remains in its early stages, and further investigations are needed.

For patients with breast reconstruction, local recurrence remains the most critical challenge. The local tumor recurrence rate for patients undergoing breast‐conserving surgery is as high as 39%. Even with adjuvant postoperative radiotherapy, the local tumor recurrence rate for patients still reaches 14%.^[^ ^4^ ^]^ In addition, current imaging techniques can only detect tumors with a cell count of more than 10^6^ malignant cells.^[^ ^5^ ^]^ Hence, the development of a novel prosthesis able to specifically sense and release therapeutic drugs at tumor sites and simultaneously reconstruct the breast for patients is of utmost importance. To achieve tumor‐specific drug delivery in tumors, hydrogel can be an ideal drug delivery system for local drug delivery and in situ cancer treatment.^[^ ^6^ ^]^ Also, the characteristics of the tumor microenvironment (TME) can be utilized for designing tumor‐specific response hydrogel.^[^ ^7^ ^]^ TME characteristics such as high reactive oxygen species (ROS) and low pH have been demonstrated to successfully trigger hydrogel degradation at tumor sites. Interestingly, ROS not only contributes to tumor development but also influences the antitumor immune response.^[^ ^8^ ^]^ And recent research has indicated that utilizing ROS‐responsive hydrogel to release therapeutic drugs at tumor sites could be an effective strategy for tumor therapy.^[^ ^9^ ^]^ Therefore, we combined this ROS‐responsive hydrogel with a 3D‐printed breast prosthesis to create a tumor‐specific drug‐delivery prosthesis.

Ferroptosis is a recently discovered form of cell death that occurs via iron‐dependent cell membrane lipid peroxidation.^[^ ^10^ ^]^ Recent studies have shown that breast cancer is particularly susceptible to ferroptosis.^[^ ^11^ ^]^ Ferroptosis‐inducing drugs have been found to effectively suppress malignant tumors, including breast cancer, and to enhance the anti‐tumor immune response.^[^ ^12^ ^]^ RSL3 is a small molecule inducer of ferroptosis that inhibits the synthesis of glutathione peroxidase 4 (GPX4), which is the major phospholipid hydroperoxide (PLOOH)‐neutralizing enzyme. Inactivation of GPX4 leads to the accumulation of lipid peroxides, which destroys the cell membrane and ultimately causes cell death.^[^ ^12^, ^13^ ^]^ Previous studies have reported that RSL3 could successfully inhibit the development of breast cancer.^[^ ^14^ ^]^ However, it remains unclear whether RSL3 encapsulated in hydrogels can also effectively suppress breast cancer and exert anticancer effects in a sustained release manner.

**Figure 1:**
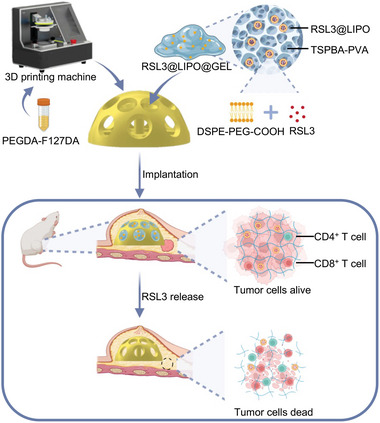
Schematic illustration showing the preparation of smart custom‐made breast prosthesis and its application in breast reconstruction and treatment for recurrence.

In our previous study, Pluronic F127 diacrylate (F127DA) was mixed with polyethylene glycol diacrylate (PEGDA) (namely, PEGDA‐F127DA), which exhibits excellent printability and mechanical properties.^[^ ^15^ ^]^ In this paper, we used PEGDA‐F127DA to serve as an ideal material for fabricating personalized shape prostheses. Inside the prosthesis, liposome‐wrapped RSL3 (RSL3@LIPO) was encapsulated into ROS‐responsive hydrogels (RSL3@LIPO@GEL) and exerted remarkable therapeutic effects on tumor recurrence. Our results demonstrated that the newly designed prosthesis not only reconstructed the breast but also smartly sensed tumors and inhibited tumor recurrence simultaneously (**Figure** **1**).

## 2 Results

### 2.1 PEGDA‐F127DA Exhibits Excellent Biocompatibility and Biostability

**Figure 2:**
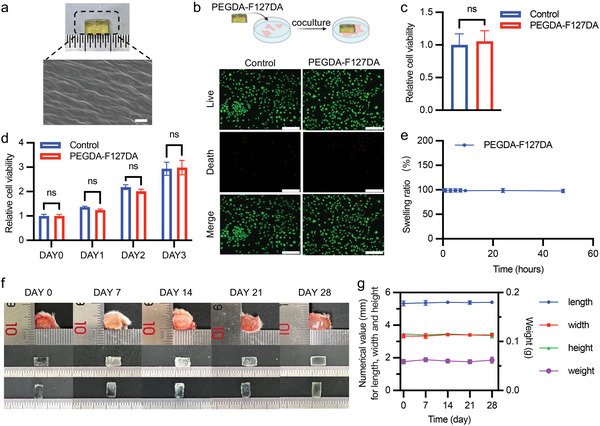
Biocompatibility and biodegradability of 3 wt% PEGDA‐15 wt% F127DA. a) SEM images of 3 wt% PEGDA‐15 wt% F127DA. Scale bar: 2 µm. b) Live/Dead fluorescence of L929 cells coincubated with 3 wt% PEGDA‐15 wt% F127DA. Scale bar: 275 µm. c) Quantification of live/dead fluorescence (*n* = 5). Student's *t*‐test, two‐tailed. ns, no significance. d) CCK‐8 assay for testing the biocompatibility of PEGDA‐F127DA (*n* = 5). Student's *t*‐test, two‐tailed. ns, no significance. e) Swelling rate of 3 wt% PEGDA‐15 wt% F127DA (*n* = 3). f,g) In vivo biostability of 3 wt% PEGDA‐15 wt% F127DA (*n* = 3).

In our previous study, we demonstrated that PEGDA‐F127DA has excellent elasticity, stability, biocompatibility, mechanical strength, and printing accuracy.^[^ ^15^ ^]^ These exceptional characteristics make PEGDA‐F127DA an ideal material for fabricating personalized prostheses. To determine the optimal concentration of PEGDA‐F127DA for fabricating the prosthesis, we first evaluated the printability and compressive modulus of F127DA hydrogels at different concentrations. We tested F127DA at 5, 10, 15, and 20 wt%. Based on printability, we excluded the 5 and 20 wt% groups as they were unprintable (Figure S1a, Supporting Information). We then compared the mechanical strength of the remaining concentrations and selected 15 wt% F127DA due to its superior mechanical properties (Figure S1b,c, Supporting Information). Next, we added PEGDA to further enhance the printability and mechanical properties of 15 wt% F127DA. We tested breast prostheses made with 15 wt% F127DA combined with 1, 3, and 5 wt% PEGDA. The results showed that prostheses with 1, 3, and 5 wt% PEGDA could be successfully printed, with higher PEGDA ratios leading to improved mechanical properties (Figure S2a–c, Supporting Information). However, biocompatibility tests revealed that cell viability was significantly higher in the 3 wt% PEGDA group compared to the 5 wt% PEGDA group (Figure S2d,e, Supporting Information). These findings suggest that a ratio of 3 wt% PEGDA and 15 wt% F127DA is optimal, balancing both biocompatibility and mechanical strength. Besides, scanning electron microscopy (SEM) revealed that the surface morphology of PEGDA‐F127DA was similar to that of commercial prostheses (**Figure** **2**a).^[^ ^16^ ^]^ To evaluate the biocompatibility of PEGDA‐F127DA, live/dead fluorescence staining, and Cell Counting Kit‐8 (CCK‐8) assays were conducted. No significant effects of PEGDA‐F127DA on cell viability were observed in either experiment, indicating that PEGDA‐F127DA exhibits excellent biocompatibility (Figure 2b–d; Figure S3, Supporting Information). Additionally, we tested the biostability of PEGDA‐F127DA via in vitro and in vivo experiments. In vitro results showed that the swelling rate of PEGDA‐F127DA was virtually unchanged (Figure 2e). Moreover, the in vivo results indicated that PEGDA‐F127DA remained up to 28 days with no significant changes in weight, length, height, or width (Figure 2f,g; Figure S4, Supporting Information, even showed PEGDA‐F127DA had no significant change on day 49). Taken together, the results indicated that PEGDA‐F127DA exhibits great biocompatibility and biostability and is suitable for fabricating breast prostheses.

### 2.2 Characterization of the Designed 3D‐Printed Prosthesis

**Figure 3:**
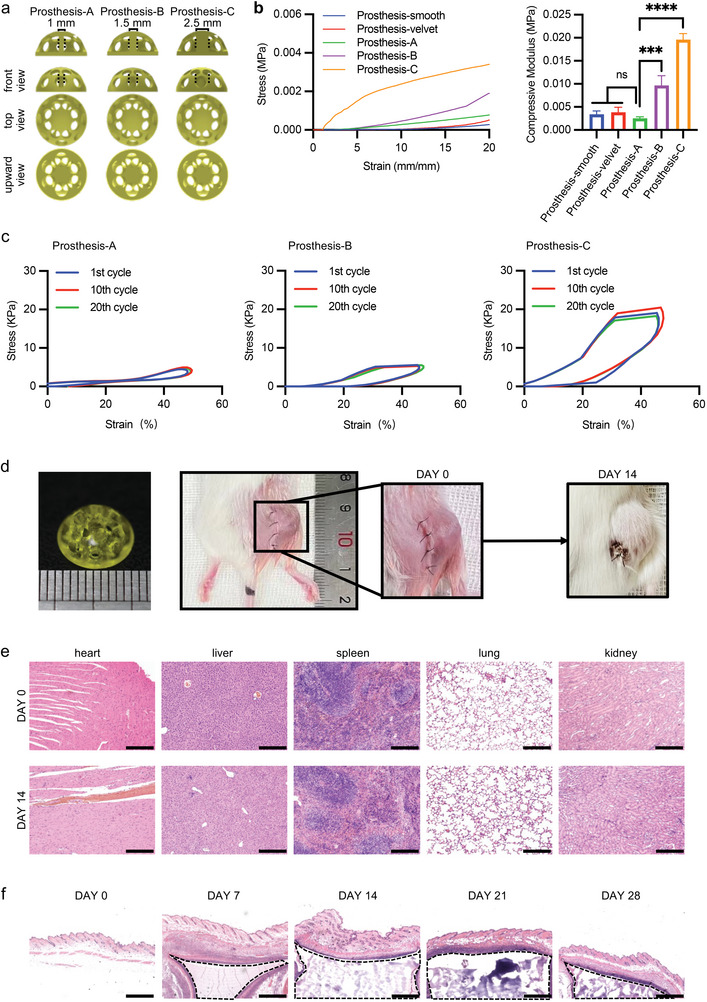
Characterization of the designed prosthesis. a) 3D‐printed models of breast prostheses with different internal columns. Internal columns are indicated with black dotted lines. b) The compressive modulus of 3D‐printed prosthesis models and commercial breast prosthesis (*n* = 3). One‐way ANOVA, two‐tailed. ns, no significance; ^*^ *p* < 0.05, ^**^ *p* < 0.01, ^***^ *p* < 0.001, and ^****^ *p* < 0.0001. c) Cyclic compression loading curves of prosthesis models (*n* = 3). d) Models of the prosthesis and its implanted effect on the appearance of mice. e) H&E staining of major organs (*n* = 3). Scale bar: 275 µm. f) H&E staining of 3 wt% PEGDA‐15 wt% F127DA‐wrapped skins (*n* = 3). Hydrogels are marked with black dashed curves. Scale bar: 650 µm.

Previous studies have indicated that the success rate of breast reconstruction is improved when prostheses contain numerous pores, as fat can grow within the prostheses.^[^ ^17^ ^]^ To fabricate a prosthesis capable of loading drugs and allowing fat tissue to grow, we designed three candidate prostheses with numerous pores and different diameters of internal columns, which were 1 mm (prosthesis‐A), 1.5 mm (prosthesis‐B) and 2.5 mm (prosthesis‐C), respectively (**Figure** **3**a). To select the most suitable prosthesis, we performed the compressive modulus test and cyclic compression test. The results showed that prosthesis‐A exhibited a compressive modulus closest to that of two commercial prostheses (smooth and velvet) (Figure 3b), suggesting that prosthesis‐A with a 1 mm diameter of the internal column could be a good candidate for breast reconstruction. And the repetitive cyclic compression loading curves of prosthesis‐A, prosthesis‐B, and prosthesis‐C showed identical paths, indicating that these prostheses have great flexibility and recoverability after 20 cycles of compression (Figure 3c). Then, we subcutaneously implanted prosthesis‐A and monitored the surgical area. After 14 days, no adverse reactions were observed, and prosthesis‐A remained intact (Figure 3d). Moreover, H&E staining of major organs (heart, liver, spleen, lung, kidney) and skin from mice implanted with prosthesis‐A showed no obvious toxic effects, indicating that prosthesis‐A exhibited excellent biocompatibility (Figure 3e,f).

### 2.3 Synthesis and Characterization of the ROS‐Responsive Hydrogel

**Figure 4:**
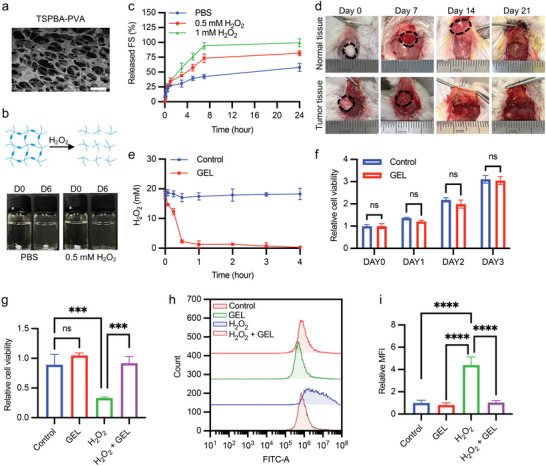
Characterization of the ROS‐responsive hydrogel. a) SEM image of ROS‐responsive hydrogel (10 wt% TSPBA‐ 5 wt% PVA). Scale bar: 20 µm. This experiment was performed thrice. b) Morphological changes in the hydrogel in PBS with or without 0.5 mmol H_2_O_2_ for 6 days. c) Cumulative release profiles of fluorescein‐contained gel incubated in PBS with or without H_2_O_2_ (*n* = 3). d) Gel degradation in normal tissue and tumor tissue. After implantation, the gel in normal tissue and tumor tissue was degraded separately at 3 weeks and 2 weeks (*n* = 3). Hydrogels are marked with black dashed curves. e) H_2_O_2_ content detection of H_2_O_2_ (20 mmol) incubated with TSPBA‐PVA (*n* = 3). f) CCK‐8 assay of L929 cells coincubated with gel (*n* = 3). Student's *t*‐test, two‐tailed. ns, no significance; ^*^ *p* < 0.05, ^**^ *p* < 0.01, ^***^ *p* < 0.001, and ^****^ *p* < 0.0001. g) CCK‐8 assay of L929 cells coincubated with gel in PBS with or without 0.5 mmol H_2_O_2_ (*n* = 3). One‐way ANOVA, two‐tailed. ns, no significance; ^*^ *p* < 0.05, ^**^ *p* < 0.01, ^***^ *p* < 0.001, and ^****^ *p* < 0.0001. h) Flow cytometry analysis of ROS levels in L929 cells treated with gel in PBS with or without 0.5 mmol H_2_O_2_ (*n* = 3). i) Quantitative analysis of ROS levels in L929 cells treated with gel in PBS with or without 0.5 mmol H_2_O_2_ (*n* = 3). One‐way ANOVA, two‐tailed. ^*^ *p* < 0.05, ^**^ *p* < 0.01, ^***^ *p* < 0.001, and ^****^ *p* < 0.0001.

The ROS‐responsive hydrogel was fabricated by crosslinking polyvinyl alcohol (PVA) with the ROS‐labile linker *N^1^*‐(4‐boronobenzyl)‐*N^3^*‐(4‐boronophenyl)‐*N*,1*N^1^,N^3^,N^3^*‐tetramethylpropane‐1,3‐diaminium (TSPBA).^[^ ^18^ ^]^ Specifically, the TSPBA linker was synthesized by reacting *N,N,N′,N′*‐tetramethyl‐1,3‐propanediamine with 4‐(bromomethyl) phenylboronic acid (Figure S5a, Supporting Information), and the linker was characterized by ^1^H Nuclear Magnetic Resonance Spectra (^1^H‐NMR) (Figure S5b, Supporting Information). To determine the appropriate proportions of TSPBA and PVA, we initially evaluated various concentrations (2.5, 5, and 10 wt%) of each component for their gel‐forming capabilities (Figure S6a, Supporting Information). The results indicated that 2.5 wt% TSPBA exhibited insufficient gel formation and was therefore excluded. By comparing the degradation and fluorescein release profiles under H_2_O_2_ conditions, we found that the hydrogel with 10 wt% TSPBA demonstrated a superior sustained‐release effect compared to 5 wt% TSPBA, leading to the selection of 10 wt% TSPBA (Figure S6b, Supporting Information). Subsequently, we tested 10 wt% TSPBA in combination with 2.5, 5, and 10 wt% PVA. The 10 wt% PVA group showed relatively weaker gel formation and was thus excluded (Figure S6a, Supporting Information). Further comparison of drug release rates revealed that 5 wt% PVA provided a better sustained‐release effect than 2.5 wt% PVA (Figure S6c, Supporting Information). Therefore, based on these findings, we selected 10 wt% TSPBA and 5 wt% PVA to prepare the ROS‐responsive hydrogel. The morphology of hydrogel was observed by SEM, which exhibited a porous structure (**Figure** **4**a). Subsequently, we examined the ROS responsiveness of the hydrogel, as the TSPBA linker can be broken by H_2_O_2_ and lead to the dissociation of the hydrogel. Hydrogels were immersed in phosphate buffer saline (PBS) or 0.5 mmol H_2_O_2_, a concentration similar to the values in the TME.^[^ ^8a^ ^]^ The results indicated that the hydrogel rapidly degraded in 0.5 mmol H_2_O_2_ solutions but was present in PBS on day 6 (Figure 4b). The release profile of fluorescein further demonstrated that the fluorescein‐loaded hydrogel released fluorescein more rapidly in H_2_O_2_ solution than in PBS only, indicating that the disassemble of hydrogel was modulated by the concentration of H_2_O_2_ (Figure 4c). Consistently, in vivo experiments demonstrated that ROS‐responsive hydrogels in tumor tissues with higher ROS concentrations degraded more rapidly than those in normal tissues. The hydrogel in normal tissue remained for up to 2 weeks, while the hydrogel at the tumor site disappeared at 2 weeks (Figure 4d). Furthermore, the H_2_O_2_ scavenging test showed that ROS‐responsive hydrogels could effectively reduce the level of H_2_O_2_ in the solution (Figure 4e). These results collectively demonstrated that the ROS‐responsive hydrogel has excellent ROS responsiveness.

Next, we performed cell viability assays to investigate the biocompatibility of the hydrogel and its ROS scavenging ability.^[^ ^19^ ^]^ The results indicated that hydrogel had no effect on cell growth (Figure 4f) and could alleviate the inhibition of H_2_O_2_ on cell growth (Figure 4g). In addition, we assessed the in vivo biocompatibility of TSPBA‐PVA. Haematoxylin and eosin (H&E) staining of major organs and skin from mice implanted with the hydrogel demonstrated no significant toxic effects, confirming the hydrogel's excellent biocompatibility (Figure S7, Supporting Information). Furthermore, 2′,7′‐dichlorodihydrofluorescein diacetate (DCFH‐DA) staining results showed that the hydrogel significantly reduced ROS levels in cells treated with H_2_O_2_‐containing solutions, indicating that the hydrogel reversed the inhibitory effect of H_2_O_2_ on cell growth by reducing its intracellular H_2_O_2_ level (Figure 4h,i).

### 2.4 ROS‐Responsive Hydrogels Loaded with Ferroptosis Inducer Can Sense and Treat Cancer

**Figure 5:**
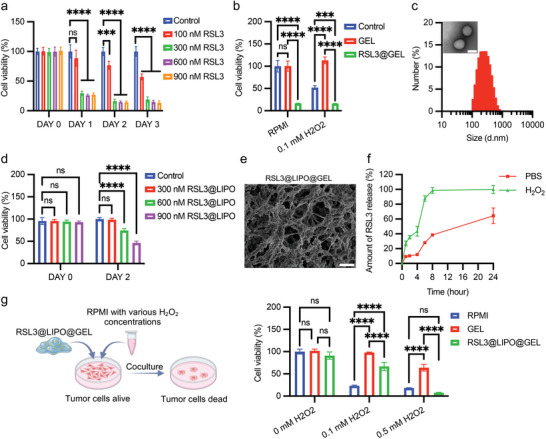
Characterization of ROS‐responsive hydrogels loaded with RSL3@LIPO. a) CCK‐8 assay of 4T1 cells treated with various concentrations of RSL3 (*n* = 5). One‐way ANOVA, two‐tailed. ns, no significance; ^*^ *p* < 0.05, ^**^ *p* < 0.01, ^***^ *p* < 0.001, and ^****^ *p* < 0.0001. b) CCK‐8 assay for analyzing proliferation of 4T1 cells treated with or without 0.1 mmol H_2_O_2_ and separately cocultured with gel and RSL3@gel (*n* = 3). One‐way ANOVA, two‐tailed. ns, no significance; ^*^ *p* < 0.05, ^**^ *p* < 0.01, ^***^ *p* < 0.001, and ^****^ *p* < 0.0001. c) The size distribution of liposomes loaded with RSL3 and its morphology was observed by transmission electron microscopy (TEM) (*n* = 3). Scale bar: 200 nm. d) CCK‐8 assay for testing toxicity of RSL3@LIPO (*n* = 3). One‐way ANOVA, two‐tailed. ns, no significance; ^*^ *p* < 0.05, ^**^ *p* < 0.01, ^***^ *p* < 0.001, and ^****^ *p* < 0.0001. e) SEM image of ROS‐responsive hydrogel loaded with RSL3@LIPO (RSL3@LIPO@GEL). Scale bar: 5 µm. This experiment was performed thrice. f) Cumulative release profile of RSL3 from RSL3@LIPO@GEL incubated in PBS with H_2_O_2_ or not (*n* = 3). g) CCK‐8 assay for testing the drug release capacity of RSL3@LIPO@GEL when incubated with PBS or H_2_O_2_ (*n* = 3). One‐way ANOVA, two‐tailed. ns, no significance; ^*^ *p* < 0.05, ^**^ *p* < 0.01, ^***^ *p* < 0.001, and ^****^ *p* < 0.0001.

RSL3, a ferroptosis inducer, could directly inactivate GPX4 and promote ferroptosis‐induced cell death in tumor cells. To determine whether RSL3 inhibits triple‐negative breast cancer (TNBC) cell growth and induces ferroptosis of TNBC cells, we performed CCK‐8 and ferroptosis‐related tests for 4T1 mammary carcinoma cells treated with RSL3. The results showed that RSL3 could inhibit cell growth in a dose‐ and time‐dependent manner (**Figure** **5**a). Meanwhile, ferroptosis‐related tests revealed that RSL3 effectively hindered the synthesis of glutathione (GSH) and triggered the accumulation of malondialdehyde (MDA), ROS, ferrous iron (Fe^2+^), and lipid peroxidation in 4T1 cells, indicating that RSL3 could induce ferroptosis in breast tumor cells (Figure S8, Supporting Information). Then, we loaded RSL3 in ROS‐responsive hydrogel (RSL3@GEL) to specifically release RSL3 under H_2_O_2_‐containing conditions. However, the CCK‐8 assay showed that RSL3@GEL greatly suppressed the viability of tumor cells even when cultured in RPMI medium (without H_2_O_2_ exposure) (Figure 5b), indicating that this small molecule drug leaks when directly loaded in the hydrogel. To overcome this, we loaded RSL3 into liposomes (RSL3@LIPO). Dynamic light scattering (DLS) showed that the size of RSL3@LIPO was uniform, with an average size of 200 nm (Figure 5c). And the encapsulation efficiency (EE) and drug loading capacity (DLC) of RSL3@LIPO were determined by high‐performance liquid chromatography (HPLC), which were 88.44 and 44.22 wt%, respectively. Moreover, the CCK‐8 assay showed that RSL3@LIPO could significantly inhibit the growth of tumor cells (Figure 5d). Subsequently, RSL3@LIPO was encapsulated in the hydrogel (RSL3@LIPO@GEL). The morphology of RSL3@LIPO@GEL was observed by SEM, which showed that numerous particles have loaded in the hydrogel (Figure 5e). And the cumulative release profile of RSL3 from RSL3@LIPO@GEL was detected by HPLC. Consistently, H_2_O_2_ solutions significantly enhanced RSL3 release rates from RSL3@LIPO@GEL (Figure 5f). More importantly, CCK‐8 results showed that RSL3 barely leaked in the setting of RSL3@LIPO@GEL after incubation in RPMI medium without H_2_O_2_ exposure (Figure 5g). Also, RSL3@LIPO@GEL could release RSL3 and effectively suppress tumor cell growth in an H_2_O_2_ concentration‐dependent manner (Figure 5g). These results demonstrated that the ROS‐responsive hydrogel showed excellent cellular compatibility and could efficiently load and release RSL3@LIPO for cancer treatment.

### 2.5 RSL3@LIPO@GEL Inhibits the Growth and Metastasis of Breast Cancer In Vivo

**Figure 6:**
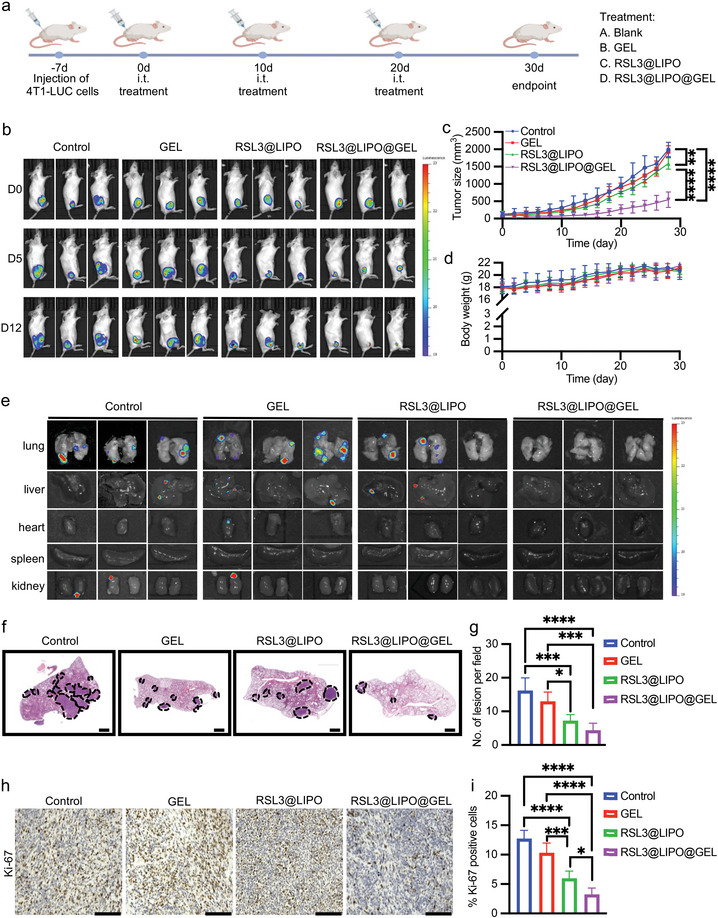
In situ formed hydrogel loaded with RSL3@LIPO for suppressing the growth and metastasis of tumors. a) Treatment schedule of 4T1 breast tumor‐bearing mice. The injections were repeated every 10 days. b) In vivo bioluminescence images of 4T1 breast tumor‐bearing mice after treatment with different formulations (*n* = 5 animals per group). c) Tumor growth curves of mice from each group (*n* = 5 animals per group). Two‐way ANOVA, two‐tailed. ^*^ *p* < 0.05, ^**^ *p* < 0.01, ^***^ *p* < 0.001, and ^****^ *p* < 0.0001. d) Body weight curves of mice throughout the whole treatment (*n* = 5 animals per group). Two‐way ANOVA, two‐tailed. ^*^ *p* < 0.05, ^**^ *p* < 0.01, ^***^ *p* < 0.001, and ^****^ *p* < 0.0001. e) Ex vivo bioluminescence images of major organs isolated from the mice. f) H&E staining images of lung tissues. Metastatic tumors are marked with black dashed curves. Scale bar: 1 mm. g) Quantification of lung metastatic lesions in H&E staining images (*n* = 5). One‐way ANOVA, two‐tailed. ^*^ *p* < 0.05, ^**^ *p* < 0.01, ^***^ *p* < 0.001, and ^****^ *p* < 0.0001. h) Histochemical staining of Ki‐67 in tumor sections. Scale bar: 125 µm. i) Quantification of the Ki‐67‐positive cells (*n* = 5). One‐way ANOVA, two‐tailed. ^*^ *p* < 0.05, ^**^ *p* < 0.01, ^***^ *p* < 0.001, and ^****^ *p* < 0.0001.

To validate the antitumor effects of RSL3@LIPO@GEL in vivo, we used the 4T1 breast tumor‐bearing mouse model. When tumor volume reached ≈100 mm^3^, the mice were intratumorally injected with normal saline, GEL, RSL3@LIPO (RSL3; 1 mg per mouse), or RSL3@LIPO@GEL (RSL3; 1 mg per mouse) (**Figure** **6**a). Tumor growth was monitored by a bioluminescence assay and digital calipers (Figure 6b,c). The unloaded hydrogel group did not show significant differences in tumor growth compared to the controls, and the RSL3@LIPO group showed a limited inhibitory effect on tumor growth. In contrast, the RSL3@LIPO@GEL group exhibited the strongest inhibitory effect on tumor growth (Figure 6b,c). Additionally, no significant difference in mouse weight was observed throughout the entire treatment (Figure 6d). In terms of metastasis, the ex vivo bioluminescence results revealed that RSL3@LIPO@GEL strongly inhibited metastasis, with no significant signal detected in the lungs and other major organs (Figure 6e). Although H&E staining revealed metastatic foci in the lungs of all mice at the end of the experiment, the number and extent of metastases were much smaller in the RSL3@LIPO@GEL group than in the other groups, indicating that drugs loaded in @LIPO@GEL exhibit a sustained release effect on cancer treatment (Figure 6f,g; Figure S9, Supporting Information). More strikingly, metastasis to other major organs was inhibited by RSL3@LIPO@GEL treatment based on H&E staining results (Figure S10, Supporting Information). In addition, immunohistochemistry (IHC) staining of the proliferation marker Ki‐67 in tumors showed that the proportion of Ki‐67 positive cells in the RSL3@LIPO@GEL treated group was significantly lower than that in the control group, unloaded hydrogel group, and RSL3@LIPO group, which were correlated with their tumor sizes (Figure 6h,i). Taken together, based on in vivo data, RSL3@LIPO@GEL could successfully suppress tumor growth and metastasis of breast cancer.

### 2.6 RSL3@LIPO@GEL Could Induce Ferroptosis in Breast Cancer and Orchestrate the TME

**Figure 7:**
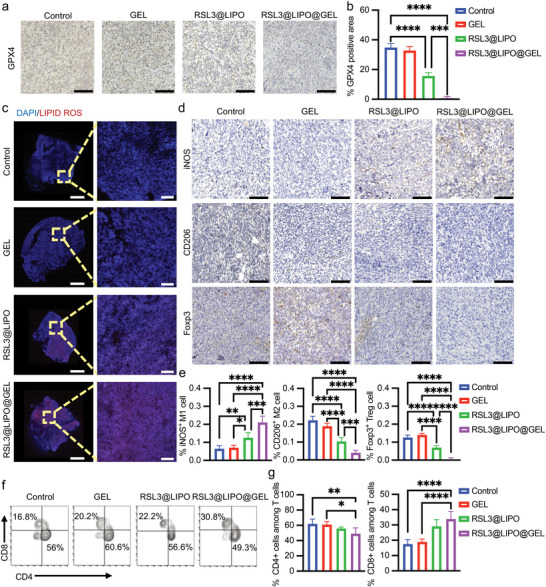
RSL3@LIPO@GEL inhibits tumor growth by eliciting ferroptosis and reversing the immunosuppressive tumor microenvironment in tumors. a) Histochemical staining of GPX4 in tumor sections. Scale bar: 125 µm. b) GPX4‐positive area of tumor tissues (*n* = 5). One‐way ANOVA, two‐tailed. ^*^ *p* < 0.05, ^**^ *p* < 0.01, ^***^ *p* < 0.001, and ^****^ *p* < 0.0001. c) Immunofluorescence staining of lipid ROS in tumor biopsies. Left‐scale bar: 2 mm. Right‐scale bar: 275 µm. d) Histochemical staining of iNOS, CD206, and Foxp3 in tumor sections. Scale bar: 125 µm. e) Quantification of M1 and M2 macrophages and Tregs in tumor sections (*n* = 5). One‐way ANOVA, two‐tailed. ^*^ *p* < 0.05, ^**^ *p* < 0.01, ^***^ *p* < 0.001, and ^****^ *p* < 0.0001. f) Flow cytometry analysis of infiltrated CD4+ and CD8+ T lymphocytes in tumor tissues. g) Quantitative analysis of CD4+ and CD8+ cells in T lymphocytes (*n* = 5). One‐way ANOVA, two‐tailed. ^*^ *p* < 0.05, ^**^ *p* < 0.01, ^***^ *p* < 0.001, and ^****^ *p* < 0.0001.

RSL3 inhibits GPX4 production and thus promotes the accumulation of lipid‐ROS, leading to ferroptosis. To explore whether tumor suppression is caused by ferroptosis, we used ferroptosis markers lipid‐ROS and GPX4 for conducting immunofluorescence staining and histochemical staining in tumor tissues from in vivo experiments. Compared to the control and RSL3@LIPO groups, RSL3@LIPO@GEL dramatically suppressed GPX4 synthesis (**Figure** **7**a,b) and greatly increased the tumor cell lipid‐ROS levels (Figure 7c). Thus, RSL3@LIPO@GEL could effectively elicit ferroptosis in breast cancer.

Previous studies indicated that the induction of ferroptosis promotes the release of calreticulin, an immunogenic cell death‐related protein, leading to enhanced engulfment of dead or dying cells by dendritic cells, more antigenic substances delivered to immune cells, and stronger antitumor immunogenicity.^[^ ^12b,c^ ^]^ To determine whether similar immunogenic effects also occurred in our model, we employed histochemical staining and flow cytometry to detect the infiltration of immune cells in tumor tissues. Relevant immune cells include tumor‐suppressive CD4+ T cells, CD8+ T cells, M1 macrophages, immunosuppressive M2 macrophages, and Tregs. The results demonstrated that the RSL3@LIPO@GEL group accumulated the most abundant M1 macrophages (Figure 7d,e) and CD4+ T and CD8+ T cells (Figure 7f,g; Figure S11, Supporting Information) in tumor tissues compared to the other groups. Additionally, compared to the other groups, the RSL3@LIPO@GEL group contained the lowest percentage of tumor‐infiltrated M2 macrophages and Tregs. The orchestration of the TME was consistent with the strongest anticancer effect of the RSL3@LIPO@GEL group. Collectively, these results suggest that RSL3@LIPO@GEL provides an effective therapeutic strategy for enhancing the antitumor immune response and inhibiting tumor development.

### 2.7 Novel Strategy for Breast Reconstruction and Treatment of Tumor Relapse

Breast cancer surgeries result in the loss of partial or entire breast tissue, which dramatically affects patients' feelings and confidence.^[^ ^20^ ^]^ Additionally, tumor recurrence remains the most critical challenge for postoperative patients. Therefore, we designed shape‐personalized therapeutic prostheses that could treat tumor recurrence for this unmet urgent need (Figure S14 Supporting Information). This therapeutic prosthesis was fabricated by incorporating a drug‐loaded hydrogel into the prosthetic structure. Impressively, the therapeutic prosthesis showed superior mechanical strength, flexibility, and recoverability (Figure S12 Supporting Information).

**Figure 8:**
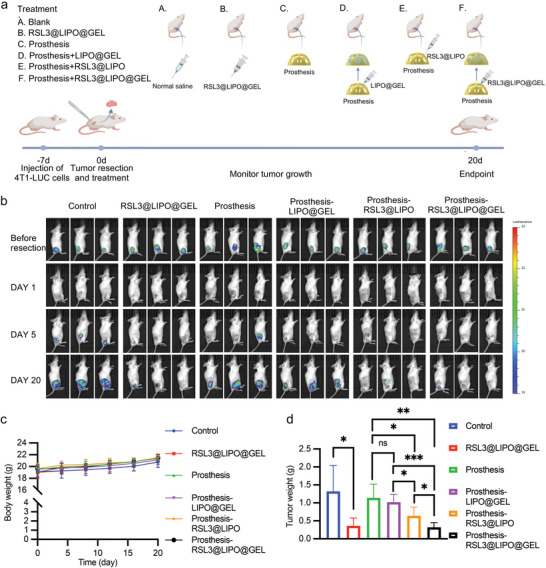
Combination of prosthesis and RSL3@LIPO@GEL to inhibit tumor growth. a) Schematic illustration of the mouse experiment. b) Representative bioluminescence images of mice treated with different compounds (*n* = 5 animals per group). c) Body weight of mice (*n* = 5). Two‐way ANOVA, two‐tailed. ^*^ *p* < 0.05, ^**^ *p* < 0.01, ^***^ *p* < 0.001, and ^****^ *p* < 0.0001. d) Tumor weight of mice (*n* = 5). Two‐way ANOVA, two‐tailed. ns, no significance; ^*^ *p* < 0.05, ^**^ *p* < 0.01, ^***^ *p* < 0.001, and ^****^ *p* < 0.0001.

In order to mimic the local tumor recurrence, we resected the tumor tissues of mice when the tumor volume reached 100 mm^3^ and left 1% residual tumor tissue behind.^[^ ^18^ ^]^ Then, different treatment strategies were carried out (**Figure** **8**a). Throughout the entire experiment, the body weights of mice barely changed, indicating that no obvious side effects occurred in each group (Figure 8c). Compared with the control group, the RSL3@LIPO@GEL group exhibited remarkable anticancer efficacy against cancer recurrence (Figure 8b,d; Figure S13, Supporting Information). More importantly, the prosthesis‐RSL3@LIPO@GEL group exhibited much lower fluorescence intensities than the prosthesis group. The tumor size and weight of the prosthesis‐RSL3@LIPO@GEL group was also smaller and less than that of the prosthesis‐RSL3@LIPO group, suggesting that the hydrogel in prostheses could exhibit a sustained‐release anticancer effect (Figure 8b,d). In sum, these results showed that our smart prostheses (prosthesis‐RSL3@LIPO@GEL group) could not only improve aesthetic appearance but also effectively treat cancer recurrence.

## 3 Discussion

In the clinic, commercial prostheses are rarely available for breast cancer patients who need partial breast reconstruction. Therefore, shape‐personalized breast prostheses are urgently needed. Here, we used PEGDA‐F127DA, which exhibited good biocompatibility, stability, and elasticity, to fabricate custom‐shaped prostheses via 3D printing (Figure S14, Supporting Information). Importantly, our results showed that the mechanical strength and surface morphology of our prosthesis are similar to those of current commercial prostheses. In sum, PEGDA‐F127DA serves as a great material to manufacture breast prostheses.

While traditional prostheses are used for breast reconstruction after whole breast resection and emerging adipose tissue engineering research explores its application in this field,^[^ ^21^ ^]^ the recurrence of tumors in patients remains a critical challenge. To improve clinical outcomes, it is important to sense and treat tumor relapse as early as possible. Therefore, breast prostheses that could sense tumor recurrence and harbor therapeutic characteristics would be intriguing. In a recent study, the application of breast prostheses loaded with drugs was investigated for the treatment of breast cancer recurrence.^[^ ^22^ ^]^ However, this prosthesis slowly released the drug in situ whether the cancer recurred or not, so tumor recurrence was only treated in the short term. Recently, ROS‐responsive hydrogels have shown prominent effects on tumor treatment by sensing ROS in tumors and specifically delivering drugs in a sustained‐release manner.^[^ ^7^ ^]^ ROS‐responsive hydrogels that release antitumor drugs in situ not only enhance drug efficacy but also minimize the side effects of systemic toxicity.^[^ ^23^ ^]^ In our study, we also demonstrated that the ROS‐responsive hydrogels, despite having no therapeutic effect on their own, were effective in encapsulating and immobilizing RSL3@LIPO at tumor sites. This effectively reduced the clearance rate of RSL3@LIPO and exhibited a sustained release effect for cancer treatment. Therefore, our breast prosthesis contains a drug‐loaded ROS‐responsive hydrogel that senses and treats tumor recurrence. In addition, although hydrogels are porous, ROS‐responsive, and biocompatible, they often fail to load small molecular drugs effectively. To prevent drug leakage or premature release, we used nanoparticles to wrap small RSL3 drug particles. Intriguingly, RSL3 loaded in the prosthesis‐RSL3@LIPO@GEL exhibited strong and long‐term anticancer effects, indicating that our smart prosthesis provides a remarkable sustained‐release effect from loaded anticancer drugs.

Immune cells in the tumor microenvironment (TME) play important roles in tumorigenesis. In the TME, cancer cells control the polarization of tumor‐associated macrophages (TAMs), which alert the proportions of TAM phenotypes during cancer progression. In the early stages, M1 macrophages are the dominant TAMs and inhibit tumor growth. Subsequently, malignant cells recruit macrophages and polarize them to M2 macrophages that promote tumor growth, metastasis, and angiogenesis, which worsens the prognosis.^[^ ^24^ ^]^ In addition, tumor‐infiltrating Tregs are correlated with immunosuppression and poor prognosis in cancer patients,^[^ ^25^ ^]^ while tumor‐infiltrating T cells are related to better prognosis.^[^ ^26^ ^]^ Recently, the role of ferroptosis in TME has been a hot topic in cancer research.^[^ ^27^ ^]^ Ferroptosis inducers could enhance antitumor immunity and inhibit tumorigenesis by regulating dendritic cells, CD8+ T cells, and Tregs.^[^ ^14a^ ^]^ However, it remains unknown whether ferroptosis inducer RSL3 encapsulated in ROS‐responsive hydrogels could induce immunogenic cell death. In our study, RSL3@LIPO@GEL markedly increased tumor infiltration of M1 macrophages, CD4+ T cells, and CD8+ T cells and significantly reduced the number of M2 macrophages and Tregs, indicating that the anticancer therapeutic effect of RSL3@LIPO@GEL was not only derived from ferroptosis of tumor cells but also from orchestration of the TME.

Nonetheless, our study has several limitations. First, only one drug, RSL3, was loaded into our smart prosthesis. Although TNBC is relatively sensitive to ferroptosis inducers, it is better to contain at least one other drug (such as chemotherapy or immunotherapy drugs) together within breast prosthesis for optimal therapeutic effects. Currently, it remains unknown whether two or more drugs can be harmoniously encapsulated in ROS‐responsive hydrogels and exert maximal anticancer effects. Second, it is unknown whether ROS‐responsive hydrogels could maintain biostability in breast prostheses for a relatively long time. Thus, further study is needed.

In summary, the incorporation of drug‐loaded ROS‐responsive hydrogel into the 3D‐printed prosthesis confers dual functionality to the novel breast prosthesis: not only allows for custom‐shaped reconstruction but also senses and treats breast cancer when relapses occur. This strategy, which improves aesthetic appearance and provides a therapeutic function, is a potentially appealing direction for breast prostheses.

## 4 Conclusion

In this study, we fabricated a novel shape‐personalized prosthesis with an RSL3@LIPO‐loaded ROS‐responsive hydrogel that reconstructs breasts, senses tumors, and releases RSL3@LIPO at the site of recurrence. Impressively, the released RSL3@LIPO elicits tumor ferroptosis and promotes an immune response that inhibits tumor development, metastasis, and recurrence.

## 5 Experimental Section

### Materials

*N,N,N″,N″*‐tetramethyl‐1,3‐propanediamine, 4‐(bromomethyl)phenylboronic acid, tetrahydrofuran, and poly(vinyl alcohol) (PVA) were all purchased from Shanghai Aladdin Biochemical Co., Ltd. (Shanghai, China). *N,N*‐dimethylformamide, PEGDA (molecular weight (MW) = 600 Da), fluorescein, and ethanol were purchased from Shanghai Macklin Biochemical Co., Ltd. (Shanghai, China). F127DA and lithium phenyl‐2,4,6‐trimethylbenzoyl phosphinate (LAP) were bought from EFL‐Tech Co., Ltd. (Suzhou, China). RSL3 was purchased from Selleck. DSPE‐PEG‐COOH (MW = 2000 Da) was bought from Shanghai ToYongBio Tech (Shanghai, China). Dimethyl sulfoxide (DMSO) and 3 wt% Hydrogen Peroxide (H_2_O_2_) were purchased from Sigma–Aldrich (Shanghai, China). Mammary Epithelial Cell Growth Medium (MEGM) was bought from Shanghai Zeye Bio‐Technology Co., Ltd. (Suzhou, China). Roswell Park Memorial Institute 1640 (RPMI 1640), Dulbecco's modified Eagle medium (DMEM), fetal bovine serum, 100X Penicillin‐Streptomycin Solution, phosphate‐buffered saline (PBS), trypsin, and Cell Counting Kit‐8 were sourced from Dalian Meilun Biotechnology Co., Ltd. (Dalian, China). Calcein‐AM/Propidium Iodide (PI) Cell Viability/Cytotoxicity Assay Kit, Reactive Oxygen Species Assay Kit, GSH, and GSSG Assay Kit, Lipid Peroxidation MDA Assay Kit were purchased from Beyotime Biotechnology Co., Ltd. (Shanghai, China). H_2_O_2_ Content Assay Kit was bought from Solarbio (Beijing, China). FerroOrange was sourced from Dojindo Laboratories (Kumamoto, Japan). 4,4‐difluoro‐5‐(4‐phenyl‐1,3‐butadienyl)‐4‐bora‐3a,4a‐diaza‐s‐indacene‐3‐undecanoic acid (C11‐BODIPY581/591) was purchased from ThermoFisher Scientific (Waltham, MA, USA). All flow cytometry‐related antibodies were purchased from Biolegend (Shanghai, China).

### Cell Lines

Mammary carcinoma cell line (4T1), mouse fibroblast cell line (L929), and mammary epithelial cell line (MCF‐10A) were purchased from the Chinese Academy of Sciences. 4T1‐luciferase (4T1‐luc) cells were acquired by infecting 4T1 cells with recombinant lentiviruses expressing luciferase. 4T1 cells and 4T1‐luc cells were cultured in RPMI 1640 supplemented with 10% fetal bovine serum, penicillin (100 U mL^−1^), and streptomycin (100 U mL^−1^). L929 cells were cultured in DMEM supplemented with 10% fetal bovine serum, penicillin (100 U mL^−1^), and streptomycin (100 U mL^−1^). MCF‐10A cells were cultured in MEGM supplemented with penicillin (100 U mL^−1^) and streptomycin (100 U mL^−1^).

### Mice

Five‐week‐old female BALB/C mice were purchased from Shanghai SLAC Laboratory Animal Co., Ltd. (Shanghai, China). All experimental mice were maintained in specific pathogen‐free conditions. All the solutions used in mouse experiments were sterile filtered through 0.22 µm filters (Millipore, PES). Mice were sacrificed by CO_2_ asphyxiation. All mouse experiments were approved by the Animal Ethical and Welfare Committee of Zhejiang Provincial People's Hospital (A20220006).

### Synthesis of the ROS‐Responsive Hydrogel (TSPBA‐PVA)

*N,N,N′,N′*‐tetramethyl‐1,3‐propanediamine (0.1 g, 0.75 mmol) and 4‐(bromomethyl) phenylboronic acid (0.5 g, 2.3 mmol) were added to dimethylformamide solvent (10 mL) and stirred at 60 °C overnight. Afterward, 100 mL of tetrahydrofuran (THF) was added to the mixture, and the solution was filtered and washed with THF three times.^[^ ^9^, ^18^ ^]^ Then, the mixture was dried in a vacuum oven to obtain pure TSPBA (0.3 g), which was characterized by ^1^H NMR. PVA was dissolved in deionized water and stirred at 90 °C overnight. TSPBA (10 wt% in deionized water, 2 mL) and PVA (5 wt% in deionized water, 2 mL) were mixed to form ROS‐responsive hydrogels (TSPBA‐PVA). The morphology of the ROS‐responsive hydrogel was characterized by SEM (Hitachi).^[^ ^21b^ ^]^

### Preparation and Characterization of RSL3@LIPO and RSL3@LIPO@GEL

DSPE‐PEG‐COOH (5 mg) was first dissolved in 10 mL of ethanol, and then RSL3 (2.5 mg) was slowly added to the solution at room temperature and magnetically stirred for 24 h. Afterward, the solvent was evaporated by a rotary dryer, and the solute was dissolved in 1 mL of phosphate‐buffered saline (PBS) to form RSL3@LIPO. A predetermined amount of RSL3@LIPO was added to 10 wt% PVA (1:1) and then mixed with 10 wt% TSPBA to form RSL3@LIPO@GEL. Afterward, the size of RSL3@LIPO was measured by DLS (Malvern), and the morphology of RSL3@LIPO and RSL3@LIPO@GEL was observed separately by TEM (Hitachi) and SEM (Hitachi).

### Cell Viability Assay

4T1 cells were cultured in 96‐well plates at a density of 3000 cells per well. RPMI‐1640 with different concentrations of RSL3 and RSL3@LIPO were then added separately to 96‐well plates.^[^ ^11^ ^]^ After incubation for 0 days, 1 day, 2 days, and 3 days, the medium was replaced with CCK‐8 test solution and incubated for 2 h. And the absorbance of each well was measured at OD450.

### Intracellular ROS, Lipid Peroxide, and Ferrous Iron assay

4T1 cells were seeded in 6‐well plates at 5 × 10^5^ cells per well and incubated overnight. Then cells were co‐incubated with 900 nmol RSL3 for 24 h. Next, detach adherent cells with trypsin, washed with PBS, and incubated with 10 µmol L^−1^ DCFH‐DA‐containing medium for 30 min at 37 °C, 5% CO_2_ in an incubator, followed by washing with PBS. Finally, the fluorescence was analyzed by flow cytometer. For lipid peroxide and ferrous iron assay, the procedures were similar to those described above, except that cells were stained separately with 10 µmol L^−1^ BODIPY 581/591 C11 and 1 µmol L^−1^ FerroOrange for 30 min.

### Intracellular MDA and GSH Measurement

4T1 cells were evenly seeded in 6‐well plates at 5 × 10^5^ cells per well and incubated overnight. Then cells were treated with 900 nmol RSL3 for 48 h. The intracellular MDA amount was measured by the Lipid Peroxidation MDA Assay Kit. Briefly, the treated cells were lysed in lysate, incubated with MDA detection solution at 100 °C for 15 min, cooled down, and centrifuged and the supernatant was measured at OD532. The intracellular GSH amount was monitored by GSH and GSSG Assay Kit. The treated cells were detached by trypsin, washed with PBS, treated with Protein Removal Reagent M Solution, rapid freeze‐thaws twice, centrifuged, and collected the supernatant. Subsequently, the supernatant was mixed with GSH assay working solution and incubated at 25 °C for 5 min, followed by mixing with NADPH solution for 25 min. Finally, the absorbance of the solution was measured at 412 nm with a microplate reader.

### In Vitro Biocompatibility of TSPBA‐PVA and PEGDA‐F127DA

4T1, L929, and MCF10A cells were separately seeded in 96‐well plates at a density of 3000 cells per well. The 10 µL TSPBA‐PVA was made by mixing 10 wt% TSPBA with 5 wt% PVA, and 5 µL PEGDA‐F127DA was made by curing PEGDA‐F127DA with UV light. Then, they were separately immersed in cell culture wells. After coincubation for 1 day, 2 days, and 3 days, the hydrogel was removed and Live/Dead cell staining with PI and calcein‐AM was performed.

### ROS Scavenging Ability of Hydrogel

L929 cells were cultured in a medium containing 0.5 mmol H_2_O_2_ or not and cocultured with hydrogel. After coincubation for 48 h, a CCK‐8 test was performed to test the viability of cells, and DCFH‐DA staining was performed to test the intracellular ROS levels.

### Releasing Profile of Fluorescein and RSL3 from Hydrogel

This experiment was performed at 37 °C. Fluorescein@GEL was made by mixing fluorescein with 10% TSPBA first and then added to 5% PVA. RSL3@LIPO@GEL was fabricated according to the above descriptions. Then Fluorescein@GEL and RSL3@LIPO@GEL were separately added into 1 mmol H_2_O_2_ and PBS to analyze the release profile of fluorescein and RSL3 from RSL3@LIPO@GEL. The amount of fluorescein in the supernatant was detected by UV–vis spectrophotometer (Thermo Fisher) and the released RSL3 was determined by HPLC (SHIMADZU) with a UV detector (230 nm).

### In Vitro Release of RSL3 and RSL3@LIPO from Hydrogel

4T1 cells were seeded into 96‐well plates at a density of 3000 cells per well. After overnight culture, the medium was replaced with RPMI‐1640 containing different concentrations of H_2_O_2_, and then 10 µL RSL3@GEL and 10 µL RSL3@LIPO@GEL containing 900 nmol RSL3 were separately added to the medium. After coincubation for 48 h, the medium and RSL3@LIPO@GEL were removed, and the CCK‐8 test was performed.

### Biodegradation of Hydrogel in Normal Tissue and Cancer Tissue

The ROS‐responsive hydrogel (10 wt% TSPBA‐5 wt% PVA) was implanted subcutaneously in healthy mice and tumor‐bearing mice. After implantation, the hydrogel was taken at 0 days, 4 days, 7 days, 14 days, 21 days, and 28 days to evaluate the size of the hydrogel.

### Preparation of Breast Prosthesis

Different structures of the prosthesis were designed using 3D Builder and exported to STL format. F127DA and PEGDA were dissolved in PBS containing 0.2 wt% photoinitiator LAP to form 3 wt% PEGDA‐15 wt% F127DA. The STL documents were imported into a 3D printing machine, and 3 wt% PEGDA‐15 wt% F127DA was poured into the material trough of the 3D printing machine. Afterward, the layer thickness was set at 100 µm, the light intensity was set as 16 mW cm^−2^, and the exposure time was adjusted to 4 s to print the scaffold of the breast prosthesis.

### Preparation of Breast Prosthesis Loaded with ROS‐Responsive Hydrogel

The breast prosthesis was printed with 3 wt% PEGDA‐15 wt% F127DA. Then, the prosthesis was placed in a polydimethylsiloxane (PDMS) mold with a hemispheroid hole, and TSPBA and PVA were separately injected into the pores of the prosthesis by syringes to fabricate a prosthesis loaded with ROS‐responsive hydrogel.

### Mechanical Properties of the Prosthesis

To perform the compressive modulus test, the different structures of the prosthesis were printed 10 mm in diameter and 4.8 mm in height. Then, the prosthesis was placed at the center of the platform with a compression rate of 0.5 mm min^−1^. The compression was stopped when the compression distance reached 80% of the initial height. Afterward, force and deformation data were recorded and calculated to draw a curve for the stress and strain. The linear region of this curve was used to calculate the compressive modulus of the prosthesis. To perform the cyclic compression test, the prostheses were compressed up to 45% of their initial height and the compression was released at a rate of 5 mm min^−1^ until there was no force. After the successive cycles of compressions, the cyclic compression curves were recorded.^[^ ^21b^ ^]^

### Biocompatibility and Biodegradation of PEGDA‐F127DA In Vivo

3 wt% PEGDA‐15 wt% F127DA material was used to print a cuboid with a length of 3 mm, a width of 3 mm, and a height of 5 mm. Then, these cuboids were implanted subcutaneously in healthy mice. To test the biocompatibility, the implanted cuboids were taken out, and the surrounding skins on the cuboids were excised and collected for histochemistry. Meanwhile, the length, width, and height of implanted cuboids were measured by digital calipers to perform the biodegradation test.

### Swelling Ratio of PEGDA‐F127DA

The weight of hydrogel (PEGDA‐F127DA) was determined immediately after photocuring to calculate its initial weight (W_0_). Subsequently, they were immersed in PBS at room temperature. The hydrogels were removed at different time points, dried with paper, and weighed (W_s_). Afterward, W_s_ from different time points were divided by W_0_ to calculate the swelling ratio of the hydrogel.

### In Vivo Inhibition of Primary Tumor Growth and Metastasis

A breast cancer model was established by subcutaneously injecting 1 × 10^7^ 4T1‐luc cells into the right flank of BALB/C mice. When the size of the tumor reached 100 mm^3^, the mice were randomly divided into four groups. The mice were intratumorally injected with different formulations, including normal saline, unloaded hydrogel, 50 mg kg^−1^ RSL3@LIPO, and hydrogel containing 50 mg kg^−1^ RSL3@LIPO every 10 days. After the first treatment, the tumor size was measured by a digital caliper every 2 days, and the volume of the tumors was calculated according to the following formula: (long diameter × short diameter2)/2. The progression and metastasis of the tumor were monitored by an IVIS Lumina imaging system (Perkin Elmer Ltd). At the end of the mouse experiment, the mice were sacrificed by CO_2_ asphyxiation, and their tumors and organs were fixed in paraffin or frozen at 80 °C.

### In Vivo Suppression of Tumor Relapse

A total of 1 × 10^7^ 4T1‐luc cells were subcutaneously injected into the right flank of BALB/C female mice (6 weeks). When the tumor reached 100 mm^3^, the tumor was partially resected and left 1% of tumor tissue behind. Afterward, the mice were randomly divided into six groups. Different formulations were implanted into the surgical bed, including 100 µL normal saline, 100 µL hydrogel containing 50 mg kg^−1^ RSL3@LIPO, a blank prosthesis, a prosthesis filled with hydrogel, a prosthesis filled with 50 mg kg^−1^ RSL3@LIPO, and a prosthesis filled with hydrogel containing 50 mg kg^−1^ RSL3@LIPO. The body weights of the mice and the sizes of the tumors were measured every 2 days. The progression and metastasis of the tumor were also monitored by an IVIS Lumina imaging system. The mice were euthanized with carbon dioxide when the body weight of the mice decreased by 2 g within 2 days or the tumor size reached 1500 mm^3^.

### Flow Cytometry Analysis of Immune Cells

The composition of T cells in tumors was analyzed by flow cytometry (Agilent). A single‐cell suspension was obtained from tumors on the seventh‐day post‐treatment. The collected cells were stained with APC‐conjugated anti‐CD45, BV421‐conjugated anti‐CD3, FITC‐conjugated anti‐CD4, and PE/Cy7‐conjugated anti‐CD8 antibodies. Then, the stained cells were examined by flow cytometry and analyzed by the FlowJo software.

### Immunofluorescence Staining

At the end of the mouse experiments, the mice were euthanized, and the tumors and major organs were fixed in paraffin or frozen at 80 °C. After being sectioned, the major organ slices were stained with H&E, and the tumor slices were stained with H&E, anti‐Ki‐67, anti‐GPX4, LIPID‐ROS, and immune cell‐related antibodies (anti‐iNOS, anti‐CD206, and anti‐Foxp3).

### Statistical Analysis

All experiments were repeated at least three times, and the results are shown as mean ± standard deviation. Statistical significance was calculated by GraphPad Prism. Unpaired two‐tailed Student's *t*‐test was used to analyze the results of two groups. One‐way ANOVA and Tukey's post hoc test were performed to analyze the results of multiple groups. The threshold of statistical significance was *p* < 0.05.

## Conflict of Interest

The authors declare no conflict of interest.

## Author Contributions

L.W. and C.Y. contributed equally to this work. L.W., C.Y., Y.H., and J.W. conceived and designed the experiments. Y.H., C.Y., and J.W. supervised the research. L.W., P.Z., M.M., J.L., Z.F., L.Z., L.C., X.X., and Q.G. performed the experiments. L.W., C.Y., M.X., and J.W. analyzed the data. C.Y., X.X., Y.Z., M.X., X.F., J.Z., J.C., Y.Z., Y.Z., Y.X., L.P., Y.S., L.W., S.W., and Y.H. provided suggestions and technical support. L.W. and C.Y. wrote the manuscript. All authors discussed the results and commented on it.

## Supporting information



Supporting Information

## Data Availability

The data that support the findings of this study are available from the corresponding author upon reasonable request.
