# Supplementary material for: 3D‐Printed Breast Prosthesis that Smartly Senses and Targets Breast Cancer Relapse
Source: Adv Sci (Weinh). 2024 Sep 23;11(46):2402345. doi: 10.1002/advs.202402345 (PMC11633491; doi:10.1002/advs.202402345)
Supplement: Supplementary file 1 — Supporting Information [file ADVS-11-2402345-s001.pdf]

ADVANCED SCIENCE
Open Access

# Supporting Information

for *Adv. Sci.*, DOI 10.1002/advs.202402345

3D-Printed Breast Prosthesis that Smartly Senses and Targets Breast Cancer Relapse

*Lu Wang, Chenyang Ye*, Xiangjie Xue, Mingjun Xie, Yicheng Zhi, Xiao Feng, Pengcheng Zhao, Jichun Zhou, Mi Mi, Jinrui Li, Qinhao Gu, Ye Zhao, Jiaxin Chen, Yi Zhou, Yanan Xue, Zexin Fu, Liuyi Zhou, Lulu Chen, Lei Pan, Yi Sun, Linbo Wang, Sufan Wu, Yong He* and Ji Wang**

Supporting Information

## 3D Printed Breast Prosthesis that Smartly Senses and Targets Breast Cancer Relapse

*Lu Wang, Chenyang Ye**, *Xiangjie Xue, Mingjun Xie, Yicheng Zhi, Xiao Feng, Pengcheng Zhao, Jichun Zhou, Mi Mi, Jinrui Li, Qinhao Gu, Ye Zhao, Jiaxin Chen, Yi Zhou, Yanan Xue, Zexin Fu, Liuyi Zhou, Lulu Chen, Lei Pan, Yi Sun, Linbo Wang, Sufan Wu, Yong He** *and Ji Wang**

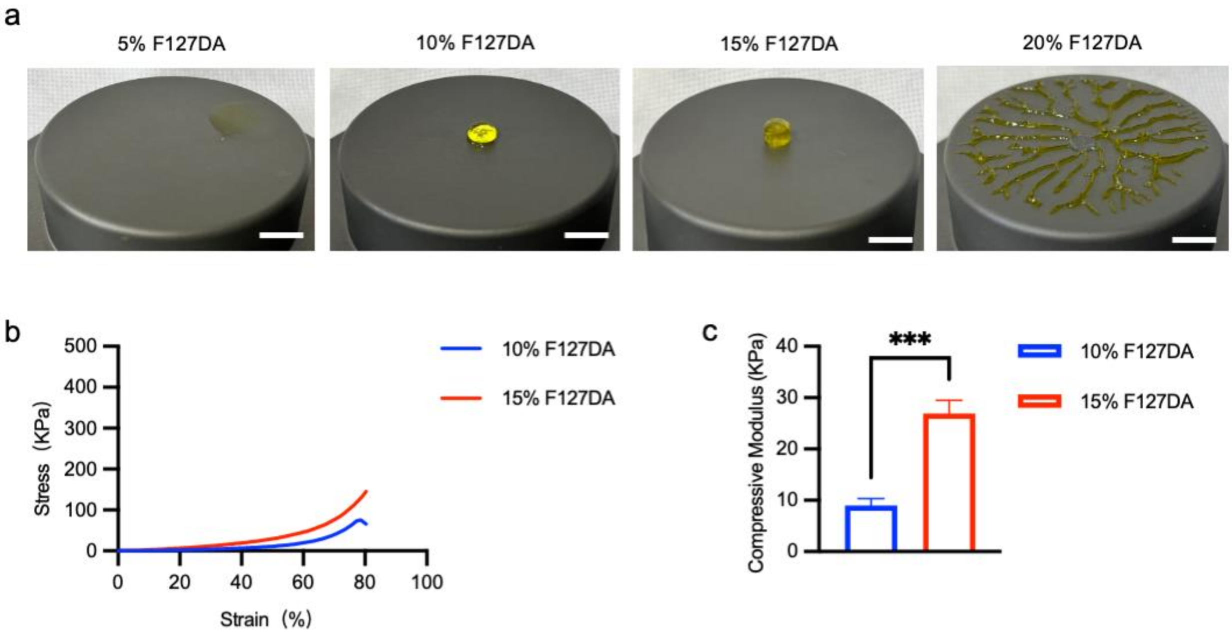


Figure S1. Characterization of F127DA hydrogels. a) Printability of F127DA hydrogels at different concentrations. Scale bar: 1 cm. b, c) Compressive stress-strain curve and compressive modulus of F127DA hydrogels at different concentrations (*n*=3). Student's t-test, two tailed. *P < 0.05, **P < 0.01, ***P < 0.001, ****P < 0.0001.

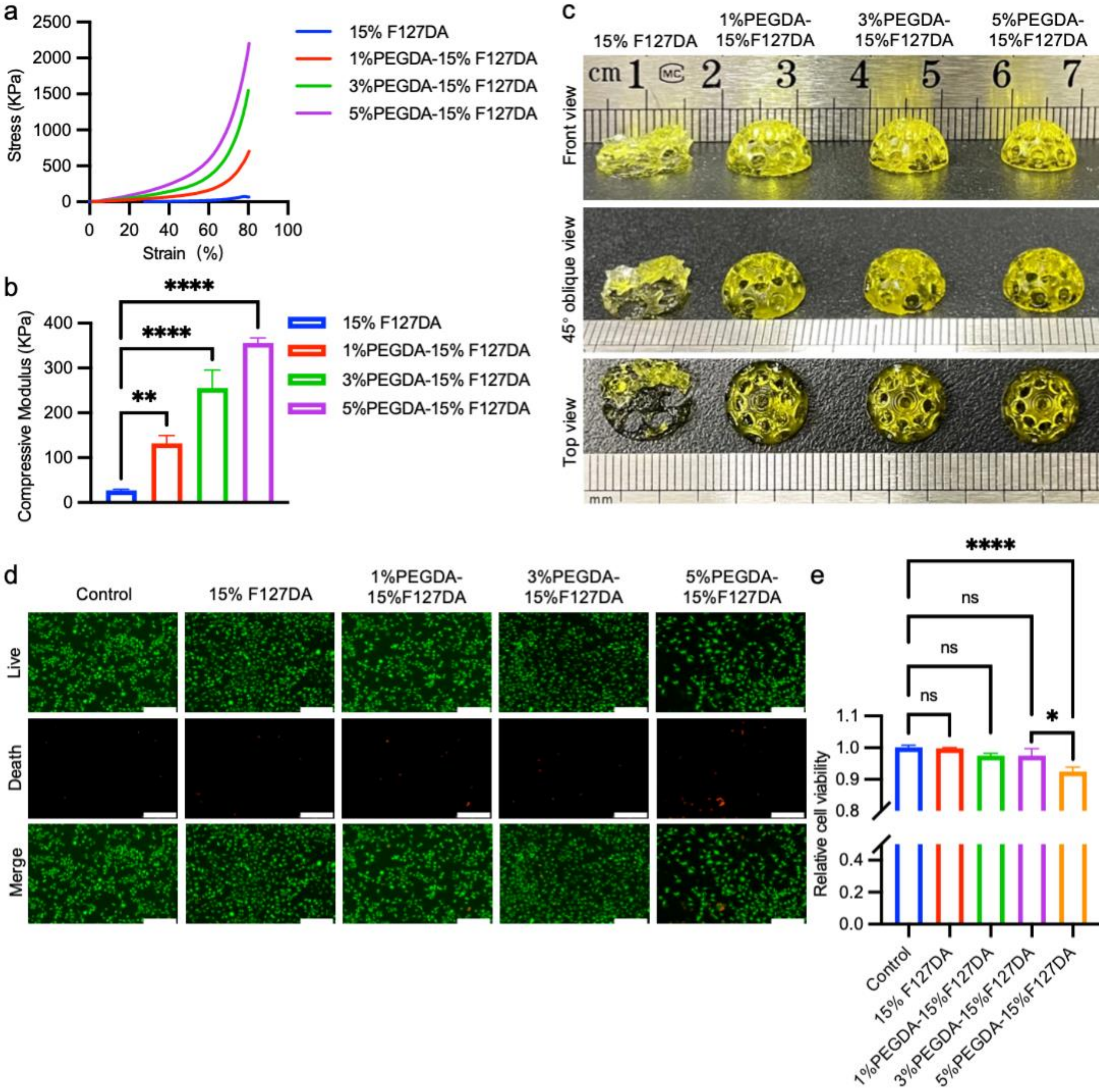


Figure S2. Characterization of PEGDA-F127DA hydrogels. a, b) Compressive stress-strain curve and compressive modulus of PEGDA-F127DA hydrogels at different concentrations (*n*=3). One-way ANOVA, two tailed. *P<0.05, **P<0.01, ***P<0.001, ****P<0.0001. c) Printability of PEGDA-F127DA hydrogels at different concentrations. d) Live/Dead fluorescence of L929 cells coincubated with PEGDA-F127DA hydrogels at different concentrations. Scale bar: 275 μm. e) Quantification of live/dead fluorescence (*n*=3). One-way ANOVA, two tailed. ns, no significance; *P<0.05, **P<0.01, ***P<0.001, ****P<0.0001.

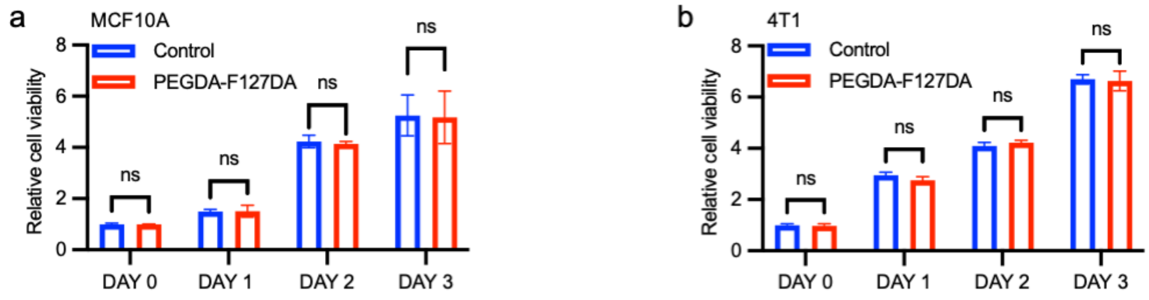


Figure S3. Biocompatibility of PEGDA-F127DA in breast epithelial cells (MCF10A) and breast cancer cells (4T1). a, b) Relative cell viability of MCF10A and 4T1 cells coincubated with 3 wt % PEGDA-15 wt % F127DA (*n*=3). Student's t-test, two tailed. ns, no significance.

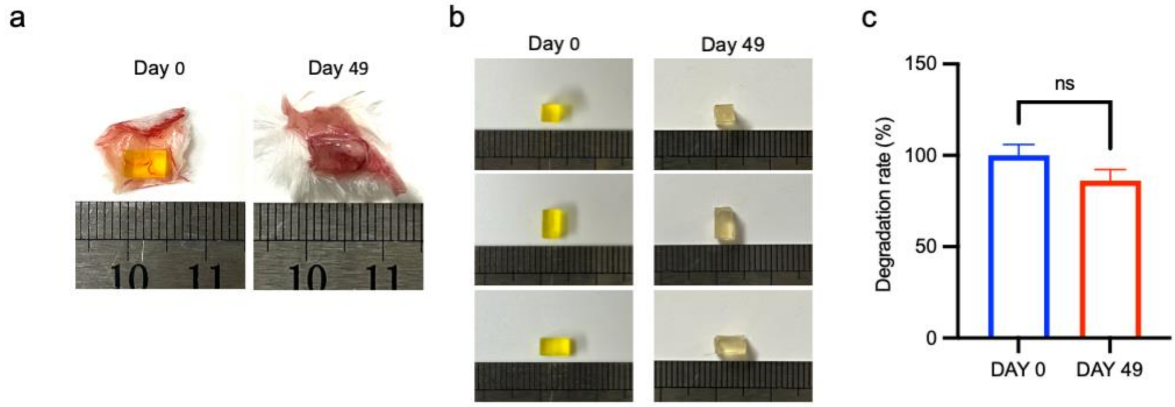


Figure S4. In vivo biostability of 3 wt% PEGDA-15 wt% F127DA on day 49. a, b) Overall morphology of PEGDA-F127DA. c) Weight of PEGDA-F127DA after freeze drying (*n*=3). Student's t-test, two tailed. *P<0.05, **P<0.01, ***P<0.001, ****P<0.0001.

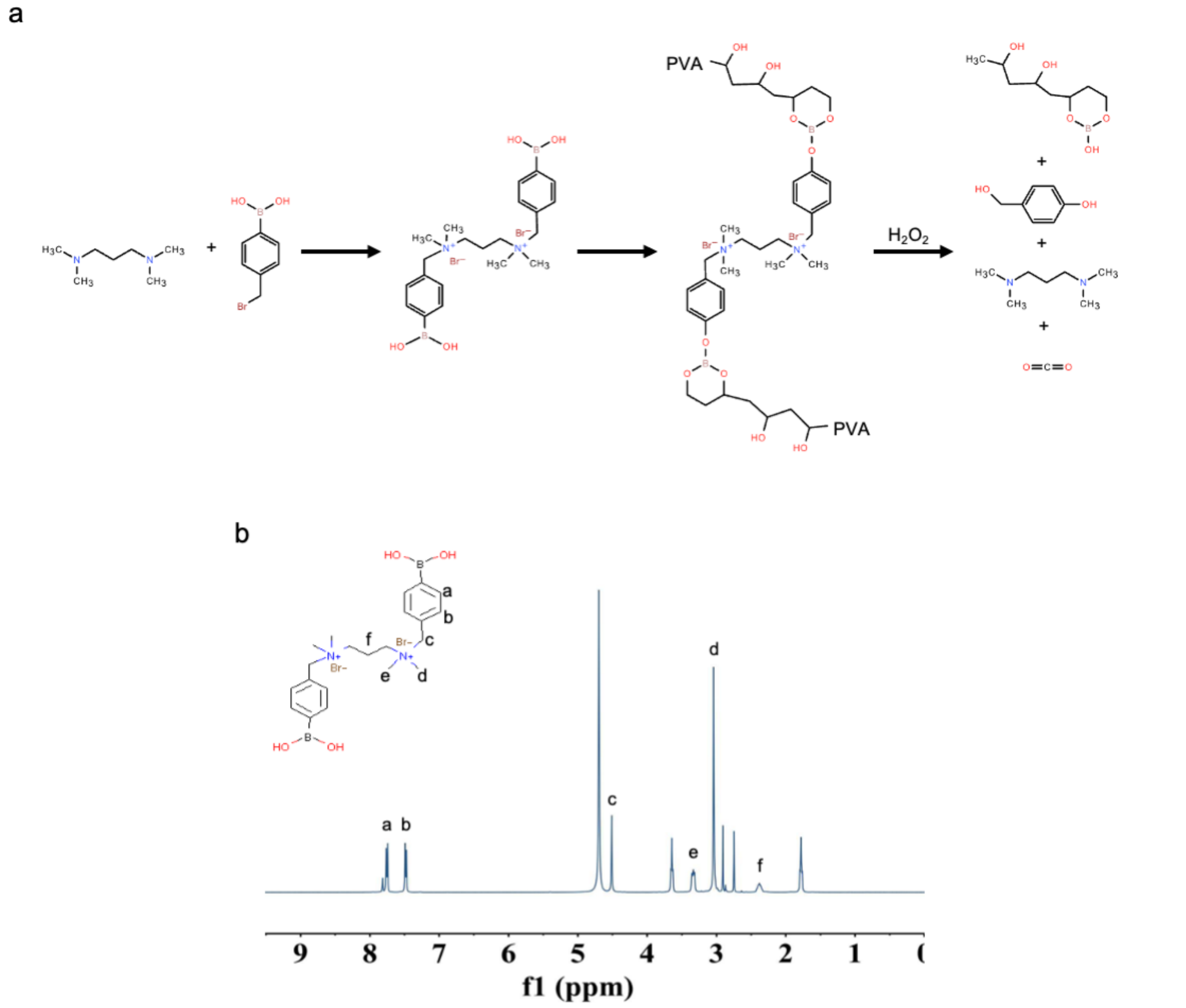


Figure S5. a) Synthesis route of TSPBA-PVA and $H_2O_2$ responsiveness mechanism of TSPBA-PVA. b) 1H-NMR (400 MHz, in D2O) spectrum of TSPBA.

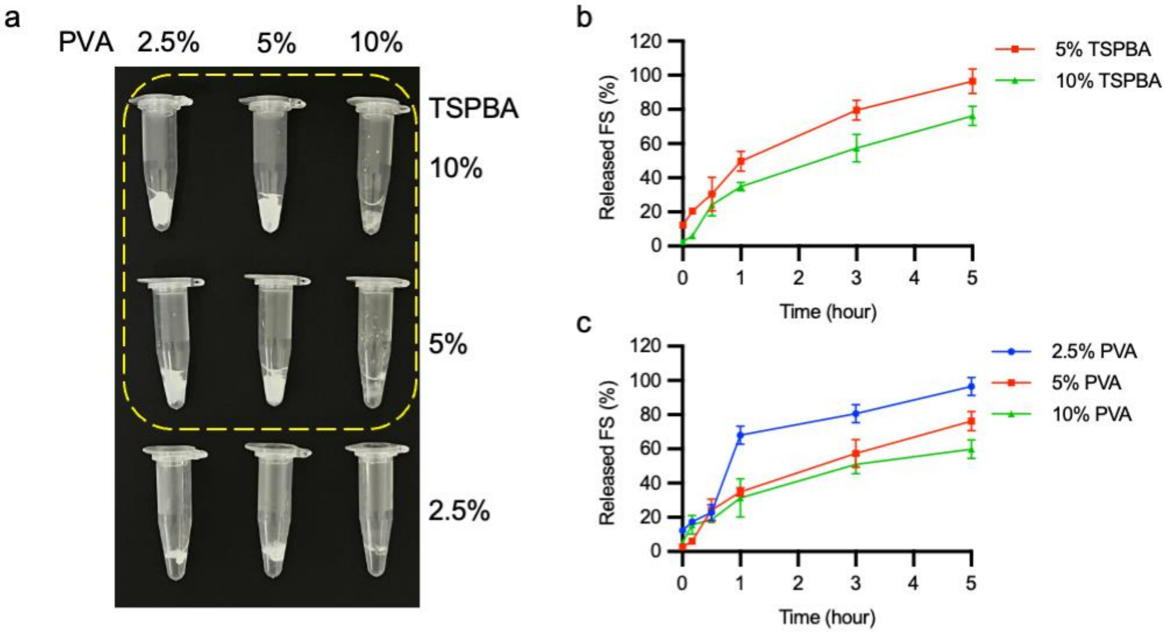


Figure S6. Characteristics of ROS-responsive hydrogel. a) ROS-responsive hydrogel formed with various concentrations of TSPBA and PVA. b) Cumulative releasing curves of fluorescein-contained gel that are formed with different concentrations of TSPBA and 5% PVA, and treated with 1 mM $H_2O_2$ ($n$=3). c) Cumulative releasing curves of fluorescein-contained gel that are formed with different concentrations of PVA and 10% TSPBA, and treated with 1 mM $H_2O_2$ ($n$=3).

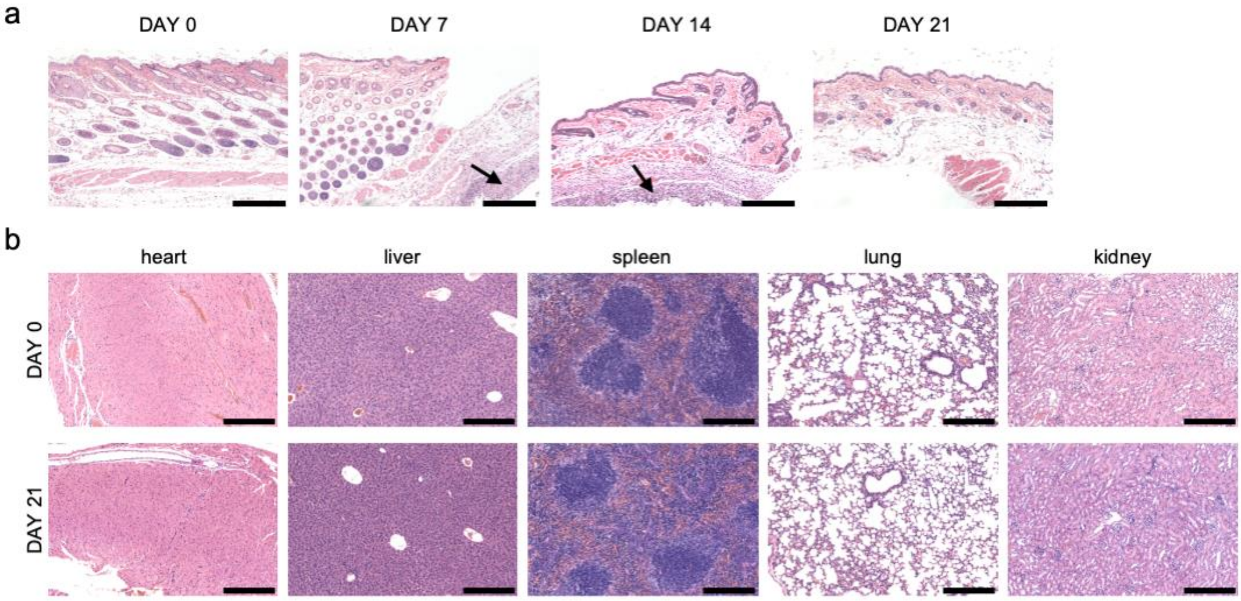


Figure S7. Biocompatibility of the ROS-responsive hydrogel. a) H&E staining of ROS-responsive hydrogel-wrapped skins. Hydrogels are indicated with black arrow. Hydrogels were degraded at the injection sites on day 21. Scale bar: 275 μm. b) H&E staining of major organs. Scale bar: 275 μm.

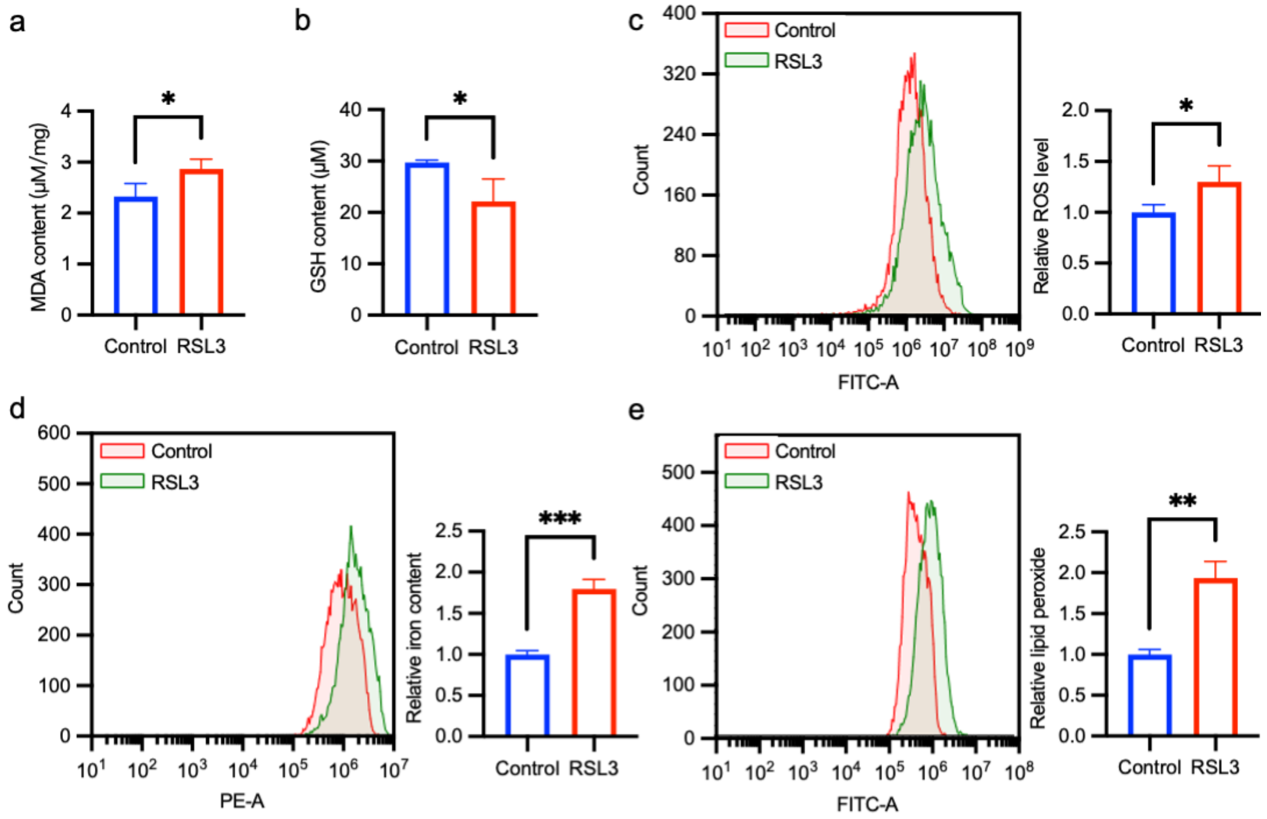


Figure S8. RSL3 efficiently triggered ferroptosis in breast tumor cells (4T1 cells). a, b) MDA and GSH content in 4T1 cells treated with RSL3 were measured (*n*=3). Student's t-test, two tailed. P value: *P<0.05, **P<0.01, ***P<0.001, ****P<0.0001. c, d, e) Flow cytometry analysis of the ROS, iron, and lipid peroxide levels in 4T1 cells treated with RSL3 (*n*=3). Student's t-test, two tailed. P value: *P<0.05, **P<0.01, ***P<0.001, ****P<0.0001.

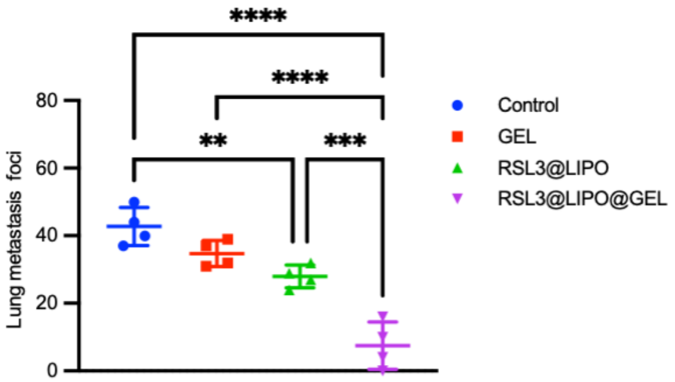


Figure S9. Numbers of lung metastasis foci in mice receiving different therapy. Lungs were taken at the end of the therapy. One-way ANOVA, two tailed. *P < 0.05, **P < 0.01, ***P < 0.001, ****P < 0.0001.

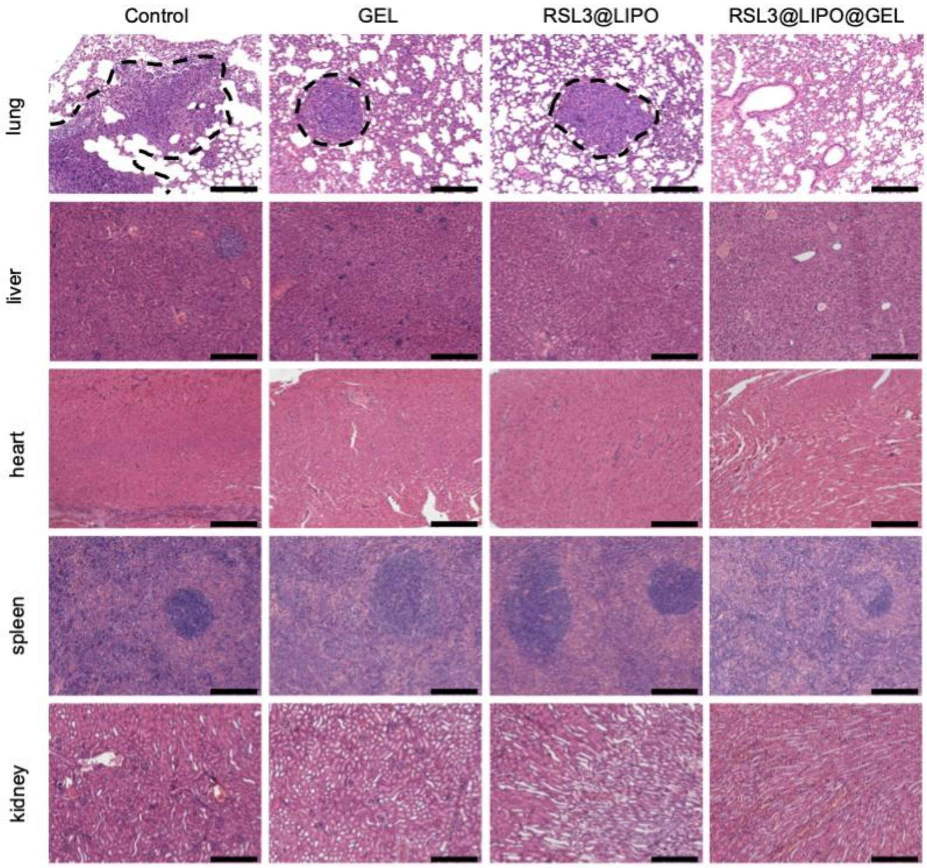


Figure S10. H&E staining of major organs. Black dashed lines indicate metastatic tumors. Scale bar: 275 µm.

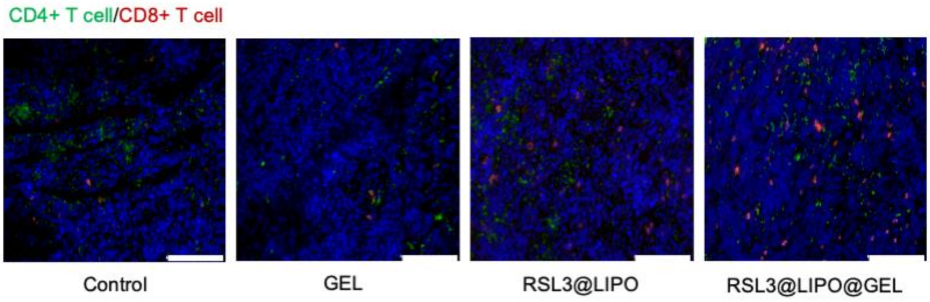


Figure S11. Immunofluorescence staining of CD4+ and CD8+ cells in tumor sections. Scale bar: 125 μm.

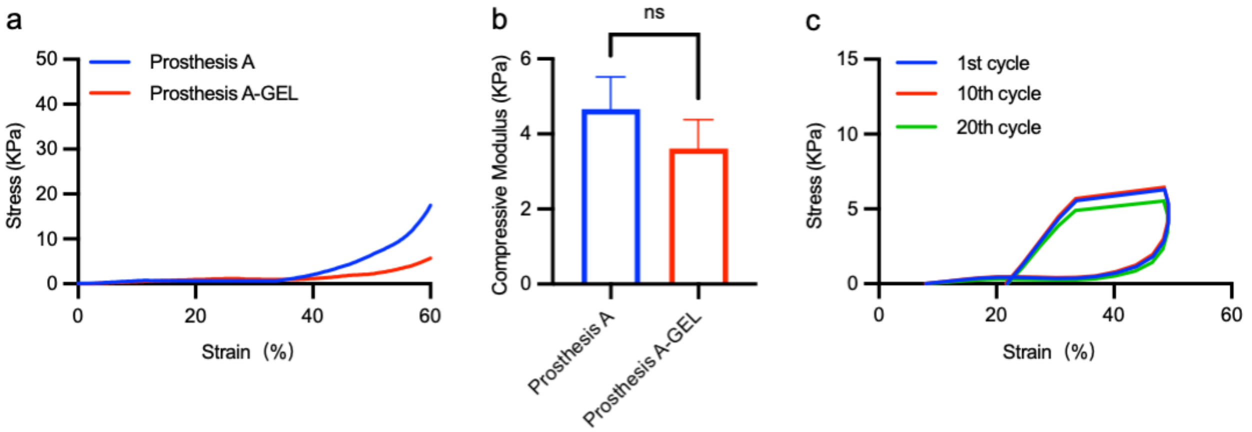


Figure S12. Characterization of Prosthesis-GEL. a, b) Compressive stress-strain curve and compressive modulus of Prosthesis with or without GEL (*n*=3). Student's t-test, two tailed. ns, no significance. c) Cyclic compression loading curves of Prosthesis-GEL.

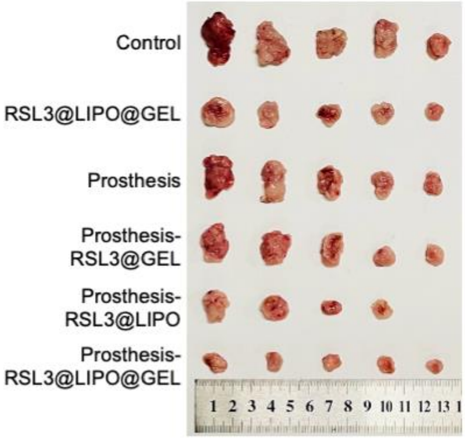


Figure S13. Subcutaneous breast tumors resected at the end of experiment (*n*=5 animals per group). One mouse in the Prosthesis-RSL3@LIPO group died during the experiment.

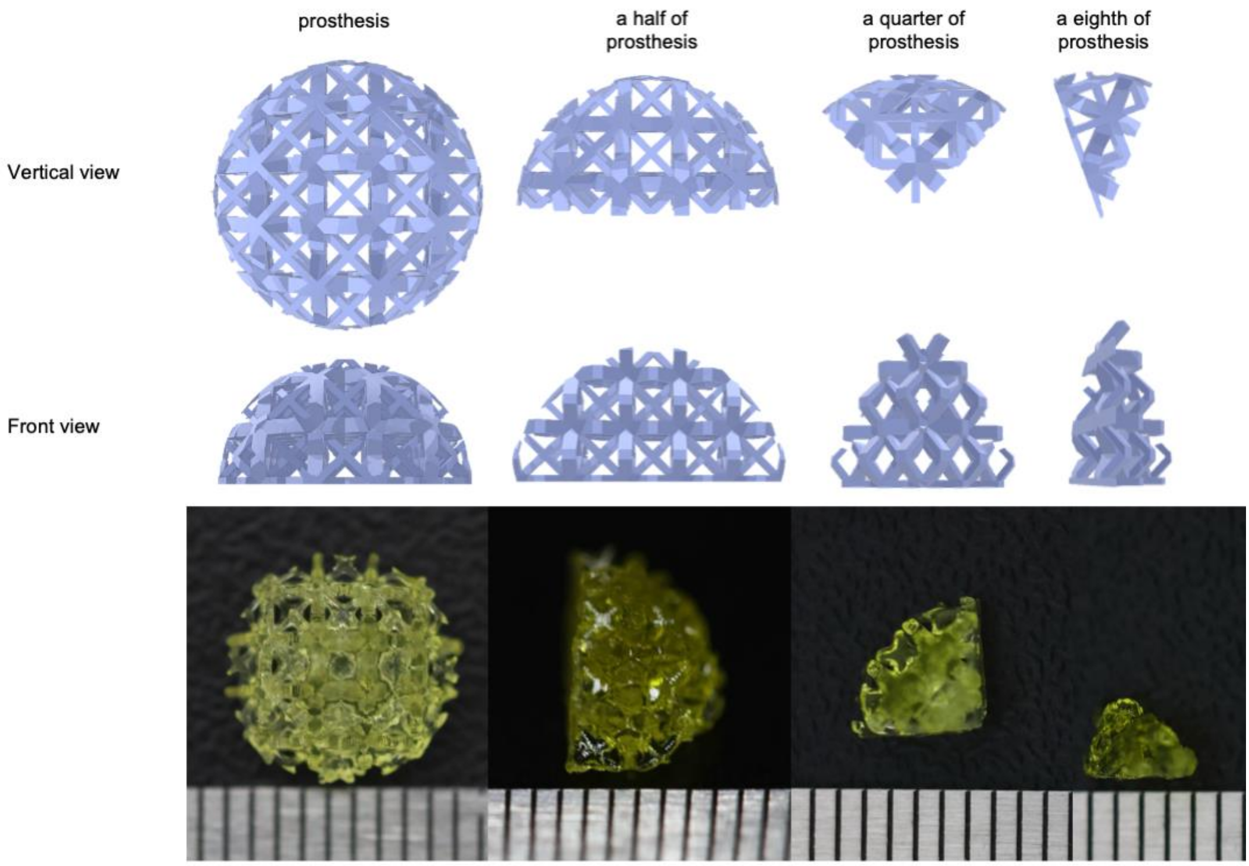


Figure S14. Models of personalized shape prostheses.
